# Diagnostic value of using ^18^F-FDG PET and PET/CT in immunocompetent patients with primary central nervous system lymphoma: A systematic review and meta-analysis

**DOI:** 10.18632/oncotarget.17456

**Published:** 2017-04-27

**Authors:** Yaru Zou, Jianjing Tong, Haiyan Leng, Jingwei Jiang, Meng Pan, Zi Chen

**Affiliations:** ^1^ Department of Dermatology, Rui Jin Hospital, Shanghai Jiao Tong University School of Medicine, Shanghai 200025, China; ^2^ Exclusive Medical Care Center, Rui Jin Hospital, Shanghai Jiao Tong University School of Medicine, Shanghai 200025, China; ^3^ Department of Hematology, Huashan Hospital, Fudan University, Shanghai 200040, China; ^4^ Department of Oncology, Huashan Hospital, Fudan University, Shanghai 200040, China; ^5^ Quintiles Asia Medical Oncology, Shanghai 200032, China

**Keywords:** ^18^F-fluorodeoxyglucose, positron emission tomography, positron emission tomography/computed tomography, primary central nervous system lymphoma, diagnosis

## Abstract

**Background:**

^18^F-fluorodeoxyglucose (^18^F-FDG) positron emission tomography (PET) and PET/CT have become two of the most powerful tools for malignant lymphoma exploration, but their diagnostic role in primary central nervous system lymphoma (PCNSL) is still disputed. The purpose of our study is to identify the usefulness of ^18^F-FDG PET and PET/CT for detecting PCNSL.

**Results:**

A total of 129 patients, obtained from eight eligible studies, were included for this systematic review and meta-analysis. The performance of ^18^F-FDG PET and PET/CT for diagnosing PCNSL were as follows: the pooled sensitivity was 0.88 (95% CI: 0.80–0.94), specificity was 0.86 (95% CI: 0.73–0.94), positive likelihood ratio (PLR) was 3.99 (95% CI: 2.31–6.90), negative likelihood ratio (NLR) was 0.11 (95% CI: 0.04-0.32), and diagnostic odds ratio (DOR) was 33.40 (95% CI: 10.40–107.3). In addition, the area under the curve (AUC) and Q index were 0.9192 and 0.8525, respectively.

**Materials and Methods:**

PubMed/MEDLINE, Embase and Cochrane Library were systematically searched for potential publications (last updated on July 16th, 2016). Reference lists of included articles were also checked. Original articles that reported data on patients who were suspected of having PCNSL were considered suitable for inclusion. The sensitivities and specificities of ^18^F-FDG PET and PET/CT in each study were evaluated. The Stata software and Meta-Disc software were employed in the process of data analysis.

**Conclusions:**

^18^F-FDG PET and PET/CT showed considerable accuracy in identifying PCNSL in immunocompetent patients and could be a valuable radiological diagnostic tool for PCNSL.

## INTRODUCTION

Primary central nervous system lymphomas (PCNSL) are extranodal malignant lymphomas that arise within the brain, eyes, leptomeninges, or spinal cord in the absence of systemic lymphoma at the time of diagnosis. The annual incidence of PCNSL in developed countries is 0.5 cases per 100,000 persons, accounting for 3–5% of primary brain tumors [1]. However, epidemiological data have shown a striking increase in the immunocompetent population over the past decades, while the incidence seems to be decreasing in patients with acquired immunodeficiency syndrome (AIDS), owning to the development of highly active antiretroviral therapy (HAART) [2–5]. Recent clinical studies have demonstrated an increase in the overall survival rates by a large margin due to the combined treatment of high-dose methotrexate-(MTX-) based chemotherapy and whole-brain radiotherapy [6]. Moreover, younger age and higher Karnofsky performance score (KPS) at the time of diagnosis are believed to be associated with prolonged survival time [7]. Accordingly, it is essential to make an early diagnosis of PCNSL and initiate early treatment correspondingly, prior to a decline of the patient's physical condition. Thus far, the diagnosis of PCNSL still relies on invasive stereotactic brain biopsy, which will inevitably be linked with a heavy expense and risk of injury. Under this circumstance, it is imperative to determine an accurate, reliable and cost-effective method for PCNSL screening.

Contrast-enhanced magnetic resonance imaging (MRI) is the standard diagnostic imaging technique when PCNSL is suspected and often shows characteristic radio-morphological features such as a lesion location adjacent to the cerebrospinal fluid (CSF) space, strong and homogenous contrast-enhancement, moderate edema and an absence of necrosis [8]. If MRI cannot be performed, then another option is contrast-enhanced computed tomography (CT), which is diagnostically equivalent in most cases [9]. The radiologic findings of MRI and CT, however, are not pathognomonic for PCNSL. Similar findings can be seen in malignant gliomas, brain metastases, and inflammatory diseases [10]. Consequently, in diagnosing PCNSL, another credible and effective radiological modality is desirable.

As a modern metabolic imaging modality, ^18^F-fluorodeoxyglucose (^18^F-FDG) positron emission tomography (PET) has shown remarkable sensitivity and specificity in the detection of systemic non-Hodgkin's lymphoma (NHL). More importantly, it has been shown to provide high accuracy in the differentiation between cerebral lymphomas and either high-grade gliomas or infectious lesions in AIDS patients [8, 11, 12]. This method alone or combined with CT (^18^F-FDG PET/CT) has been proposed as a non-invasive and accurate tool to assess disease progression in cancer patients [13, 14]. Since the tumor tissue usually has a high cellular density with an accelerated glucose metabolism, lesions of PCNSL often show a high FDG concentration. Thus, ^18^F-FDG PET and PET/CT are good at distinguishing PCNSL with hypermetabolic lesions from infection with hypometabolic lesions [15]. Furthermore, preliminary data suggest that ^18^F-FDG PET and PET/CT may be excellent in making a distinction between PCNSL and other brain tumors [16, 17]. A retrospective study by Makino et al. reported that, when it came to PCNSL and GBM with similar MRI findings, the addition of ^18^F-FDG PET could improve diagnostic accuracy compared to that with conventional MRI [18]. However, Kawai and colleagues see things differently. They found little benefit of PET to discriminate PCNSL from other neoplastic and benign diseases compared with MRI, especially for atypical lesions [19]. Unfortunately, similar studies are relatively rare, so it is difficult to draw a firm conclusion.

With the development of ^18^F-FDG PET and PET/CT, a number of studies have reported varying results about their detection ability of PCNSL. Nevertheless, the present evidence is mainly from some small sample clinical studies. Consequently, a systematic review aimed at clarifying the potential effects of ^18^F-FDG PET and PET/CT in PCNSL radiological diagnosis was essential. For this reason, we conducted a meta-analysis to evaluate the role of ^18^F-FDG PET and PET/CT in the diagnosis of immunocompetent patients with PCNSL.

## RESULTS

### Search strategy and study selection

**Figure 1:**
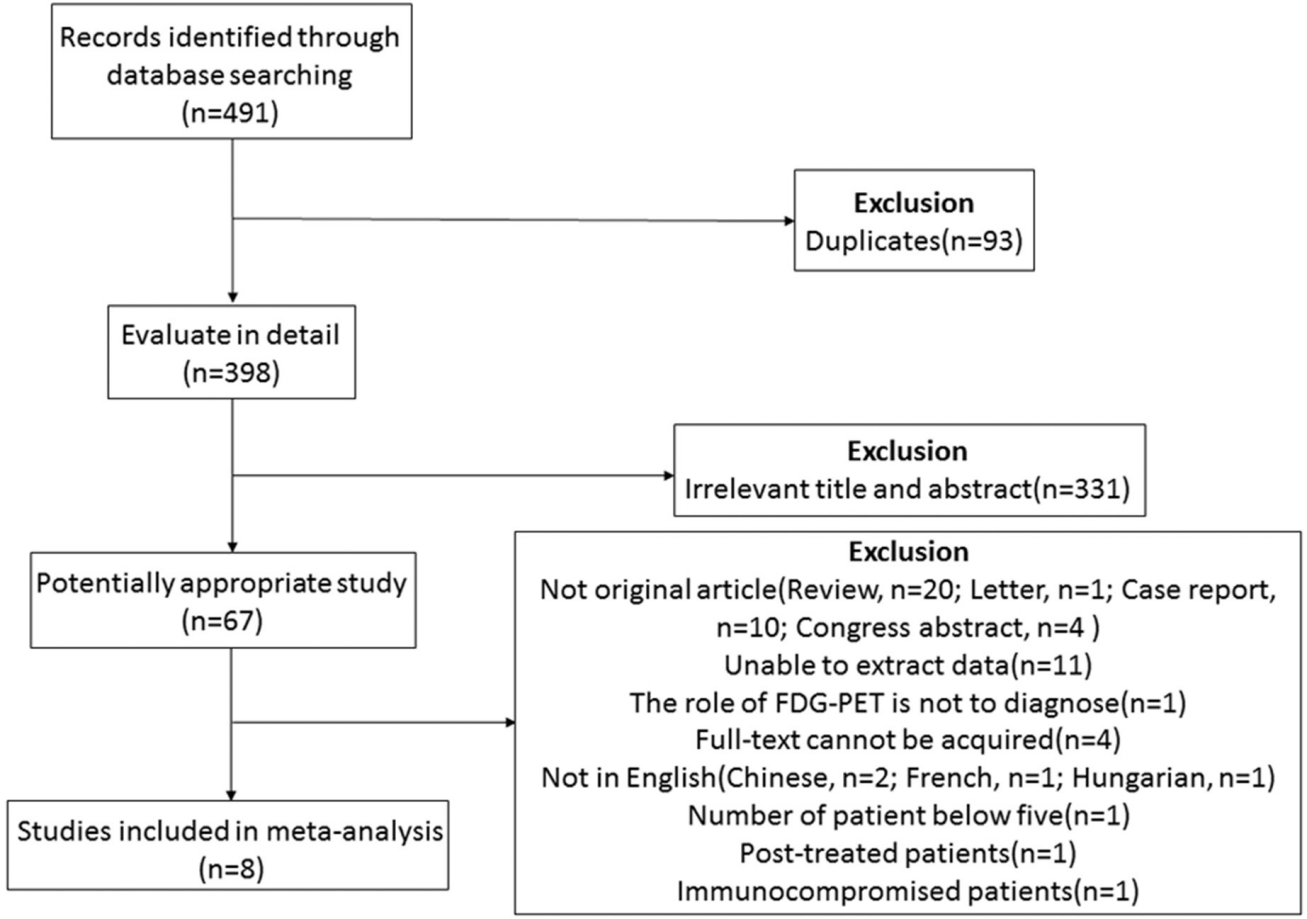
Studies evaluated for inclusion in this meta-analysis

As previously mentioned, we searched up to July 16th, 2016, which yielded a total of 491 papers: 352 in Embase, 111 in PubMed/MEDLINE, 11 in Cochrane Library, and 17 by a manual search. Among them, 93 duplicate publications were excluded. After screening for titles and abstracts, we reviewed 67 articles in detail. Of these, 59 were excluded. The exclusion criteria were as follows: (1) Articles that could not reveal the performance of ^18^F-FDG PET and PET/CT in the diagnosis of PCNSL (*n* = 1). (2) Publications without sufficient data to acquire or calculate the true-positive (TP), false-positive (FP), true-negative (TN), and false-negative (FN) (*n* = 11). (3) Publications without primary data, such as comments, letters, case reports, conference proceedings, guidelines, and reviews (*n* = 35). (4) Articles with less than five PCNSL patients enrolled (*n* = 1). (5) Articles with patients who have been treated before (*n* = 1). (6) Articles with patients who were in a situation of immunodeficiency or immunosuppression (*n* = 1). (7) Articles in which the full-text versions could not be acquired or articles published in a non-English language (*n* = 8). Finally, eight studies with a total of 129 patients were eligible for inclusion (see Figure 1).

### Data extraction and quality assessment

Independently, two investigators reviewed and extracted data from the articles. Any disagreements were resolved by discussion and a consensus. The main characteristics of the eight eligible studies are exhibited in Table 1. Most of these studies were assessed as having a low-risk of bias, according to Quality Assessment of Diagnostic Accuracy Studies-2 (QUADAS-2). Regarding the study design, all of the included studies were retrospective studies. Patients were entirely immunocompetent, and PCNSL was at least confirmed by histopathology. The patient-based diagnostic parameters of ^18^F-FDG PET and PET/CT in PCNSL from these studies are shown in Table 2. Among them, four studies used only ^18^F-FDG PET, three studies used PET/CT, and only one study used both techniques. Additionally, the lesion-based diagnostic parameters from only three studies are shown in Table 3. The sensitivity for diagnosing ranged from 87.5% to 100%. Furthermore, the highest specificity was 96.30%.

**Table 1:** Main characteristics of the included studies

Study	Country	Year	Number of patients	Sex (M/F)	Mean age	Imaging	Immune system	Study design
Palmedo et al [17]	Germany	2005	7	4/3	66.4 ± 4.9	FDG-PET	Immunocompetent	retrospective
Karantanis et al [36]	America	2007	14	10/4	58.4 ± 12.2	FDG-PET/CT	Immunocompetent	retrospective
Kosaka et al [16]	Japan	2008	34	17/17	64.2	FDG-PET, FDG-PET/CT	Immunocompetent	retrospective
Kawai et al* [19, 32]	Japan	2010	17	9/8	65.1 ± 8.7	FDG-PET	Immunocompetent	retrospective
Kawase et al* [27]	Japan	2010	6	3/3	71.8 ± 8.9	FDG-PET	Immunocompetent	retrospective
Makino et al [18]	Japan	2011	21	13/8	67	FDG-PET/CT	Immunocompetent	retrospective
Okada et al [30]	Japan	2012	18	10/8	59.3 ± 14.9	FDG-PET	Immunocompetent	retrospective
Mercadal et al [28]	Spain	2015	12	6/6	61.4 ± 12.1	FDG-PET/CT	Immunocompetent	retrospective

*Seven patients were overlapped between these two studies, therefore, we removed the data of the seven patients from the study of Kawase et al.

**Table 2:** PET in PCNSL: Patient-based data

Study	N(M/F)	TP	FP	FN	TN	Median SUVmax	Confirmation
Palmedo et al [17]	7 (4/3)	6	0	1	0	6.6 (0–10.7)	MRI, Histopathology
Karantanis et al [36]	14 (10/4)	13	0	1	0	-	MRI, Histopathology
Kosaka et al [16]	34 (17/17)	7	1	0	26	22.17 ± 5.03^a^	Histopathology, Clinical and radiologic follow-up
Kawai et al [19, 32]	17 (9/8)	13	0	4	0	12.4 (6.3–23.3)	MRI, Histopathology
Kawase et al* [27]	6 (3/3)	6	0	0	0	10.89 (8.59–20.33)	Histopathology
Makino et al [18]	21 (13/8)	14	2	0	5	–(7.9–30.5)	Histopathology
Okada et al [30]	18 (10/8)	6	1	1	10	11 (4.8–33.9)	Histopathology
Mercadal et al [28]	12 (6/6)	12	0	0	0	25 (6–39)	Histopathology

a: Mean ± SD.

*Seven patients were overlapped between the study of Kawai et al. and Kawase et al. therefore, we removed the data of the seven patients from the study of Kawase et al.

**Table 3:** PET in PCNSL: Lesion-based data

Study	Total	TP	FP	FN	TN	Median SUVmax	Confirmation
Palmedo et al [17]	9	8	0	1	0	6.6 (0–10.7)	MRI, Histopathology
Karantanis et al [36]	16	14	0	2	0	-	MRI, Histopathology
Kosaka et al [16]	34	7	1	0	26	22.17 ± 5.03^a^	Histopathology, clinical and radiologic follow-up

a: Mean ± SD.

### Heterogeneity and threshold effect assessment

**Figure 2:**
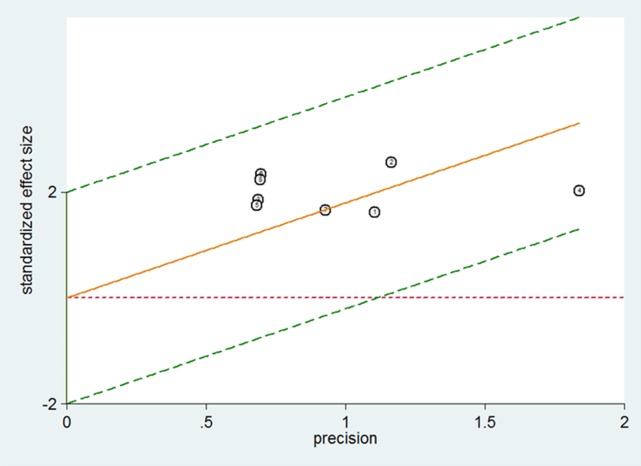
Galbraith plots of studies for the sensitivities of ^18^F-FDG PET and PET/CT in the diagnosis of PCNSL

The heterogeneity among the included studies were checked with the Chi-square test. There was no significant heterogeneity in the sensitivity (χ^2^ = 5.66, *p* = 0.5796) and specificity (χ^2^ = 7.64, *p* = 0.3656) of ^18^F-FDG PET and PET/CT in the patient-based data (see Figure 2). The Spearman correlation coefficient was –0.327 (*p* = 0.429), among the eight studies with patient-based data. This result indicated that no threshold effect existed. Thus, the Mantel–Haenszel method (fixed effects model) was adopted to estimate the pooled data.

### Diagnostic performance

**Figure 3:**
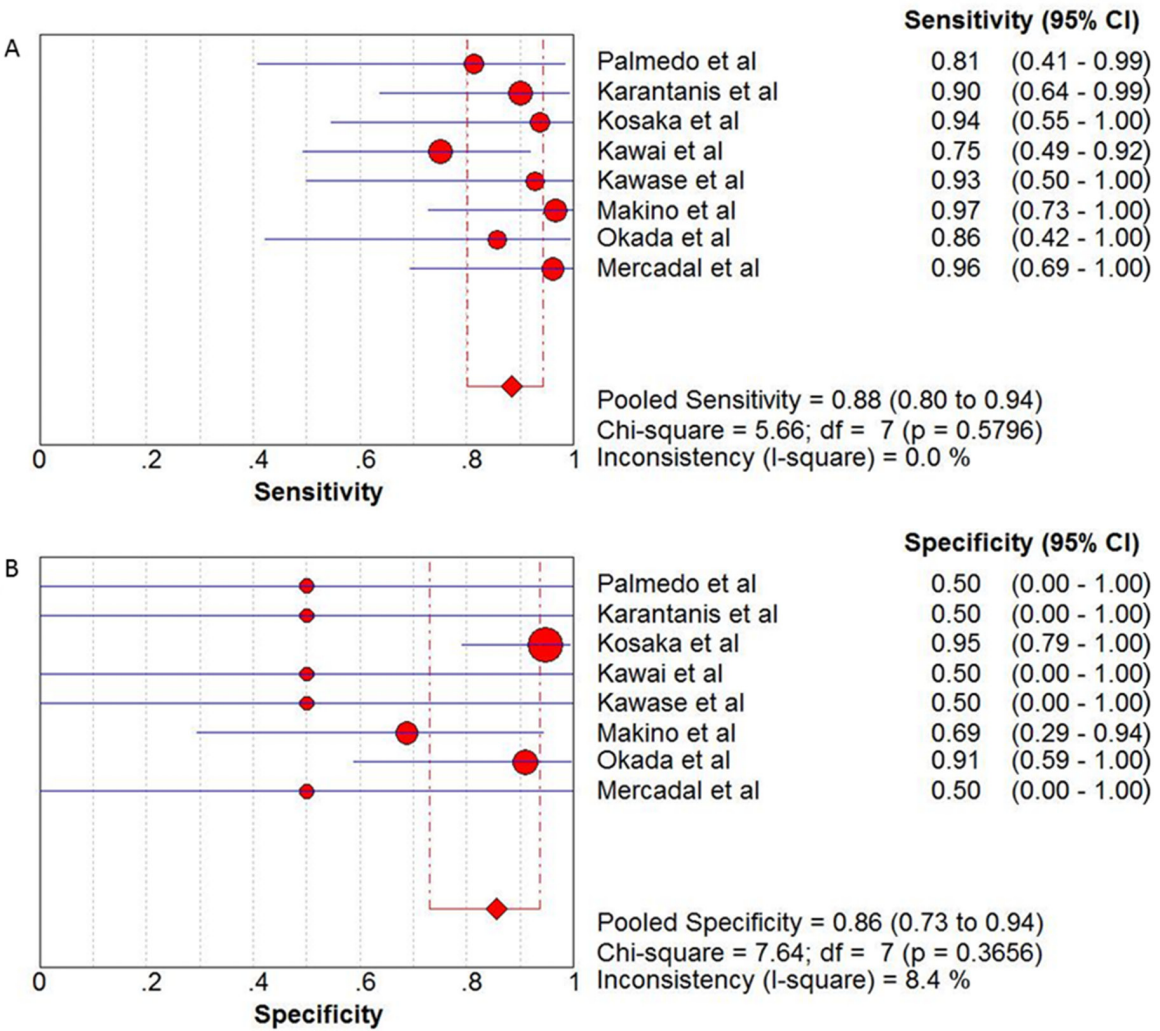
(**A**) Sensitivity and 95% confidence intervals for studies assessing the diagnostic value of ^18^F-FDG PET and PET/CT in patients with PCNSL. (**B**) Specificity and 95% confidence intervals for studies assessing the diagnostic value of ^18^F-FDG PET and PET/CT in patients with PCNSL. *The diamond represents the 95% CI of the pooled estimate.

**Figure 4:**
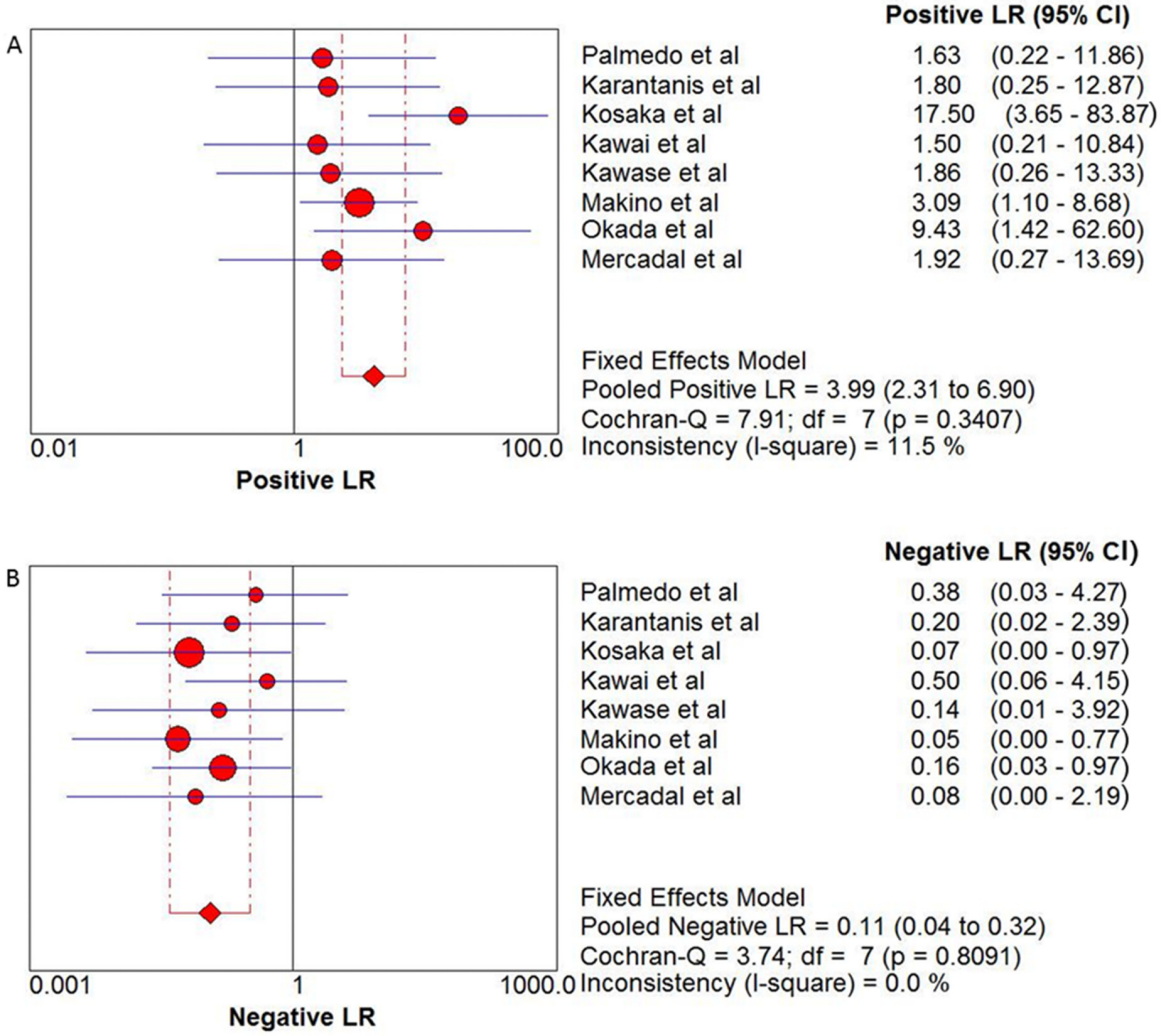
(**A**) Positive LR and 95% confidence intervals for studies assessing the diagnostic value of ^18^F-FDG PET and PET/CT in patients with PCNSL. (**B**) Negative LR and 95% confidence intervals for studies assessing the diagnostic value of ^18^F-FDG PET and PET/CT in patients with PCNSL. *The diamond represents the 95% CI of the pooled estimate.

**Figure 5:**
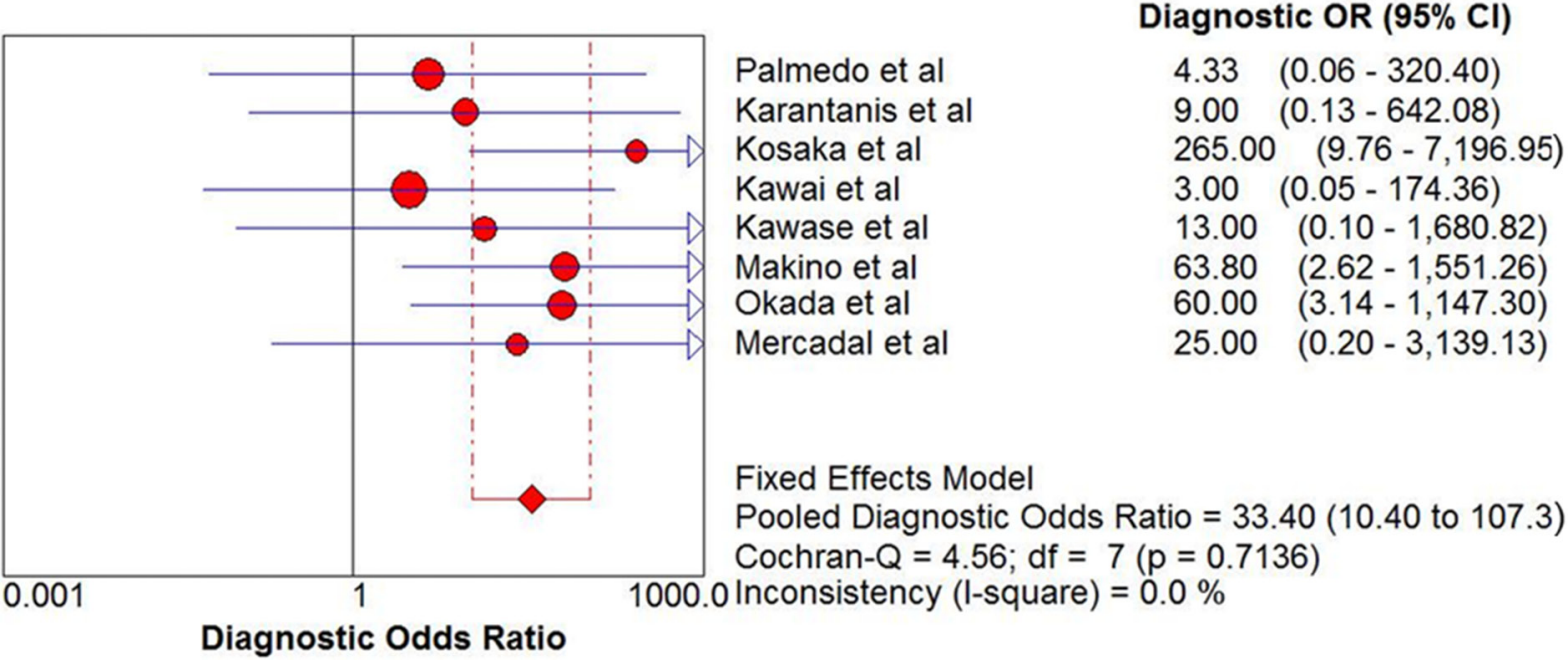
DOR and 95% confidence intervals for studies assessing the diagnostic value of ^18^F-FDG PET and PET/CT in patients with PCNSL *The diamond represents the 95% CI of the pooled estimate.

The pooled sensitivity and specificity of ^18^F-FDG PET and PET/CT in the diagnosis of PCNSL were 0.88 (95% CI: 0.80–0.94) and 0.86 (95% CI: 0.73–0.94), respectively (see Figure 3). In addition, the pooled positive likelihood ratio (PLR) was 3.99 (95% CI: 2.31–6.90) and negative likelihood ratio (NLR) was 0.11 (95% CI: 0.04–0.32) (see Figure 4). The pooled diagnostic odds ratio (DOR) was 33.40 (95% CI: 10.40–107.3) (see Figure 5).

**Figure 6:**
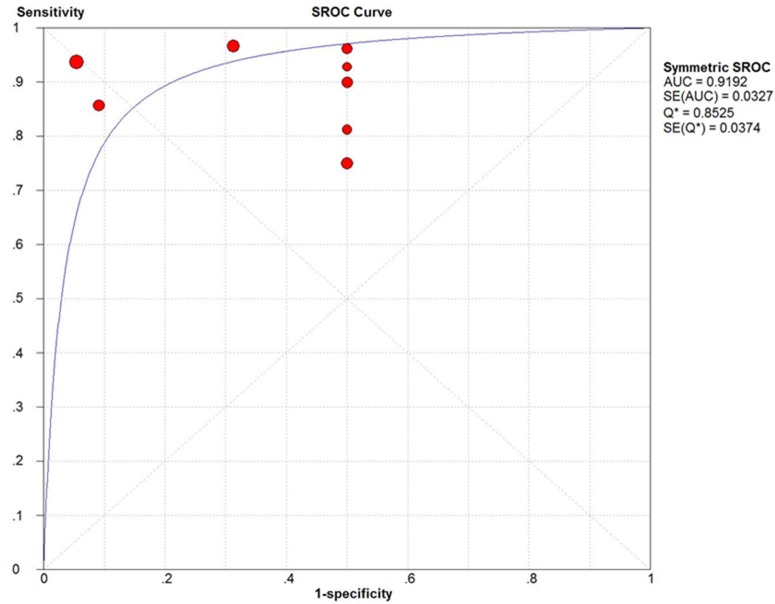
The SROC curves of ^18^F-FDG PET and PET/CT in the patient-based data of PCNSL

The summary receiver operating characteristic curve (SROC) and the Q index for ^18^F-FDG PET and PET/CT in the diagnosis of PCNSL are shown in Figure 6. The area under the curve (AUC) was 0.9192 and the Q index was 0.8525.

## DISCUSSION

PCNSL, an uncommon variant of extranodal non-Hodgkin lymphoma (NHL), can affect any part of the neuraxis, including the eyes, brain, leptomeninges, or spinal cord [20]. So far, PCNSL has been challenging to study, owing to the rarity of the disease. As a result, it was a tough task that to establish an effective diagnosis and treatment standard. Nevertheless, because PCNSL has highly aggressive tumors, both the successful treatment and improvement of prognosis would benefit from an early diagnosis.

Currently, MRI and CT are still the first-line imaging examinations used in the detection of brain lesions. Neuroimaging with cranial MRI using fluid-attenuated inversion recovery and T1-weighted sequences before and after contrast injection is the preferred method of choice for diagnosis and follow-up [21]. Studies have shown that typical findings in immunocompetent patients are homogenously enhancing single lesions (60–70% of cases) or multiple lesions (30–40% of cases) without necrosis and with a relatively small amount of edema [22]. In most cases, the possibility of PCNSL could be recognized by MRI based on these imaging signs. Still, glioblastoma multiforme (GBM), brain metastasis, and some non-neoplasm lesions with enhancement are the most likely misdiagnosed diseases [23–25]. In a dilemma, an accurate initial diagnosis is of vital importance because the management and prognosis of these diseases are quite at odds with each other. For instance, if the patient is highly suspected of having GBM, a craniotomy would be recommended. In contrast, for PCNSL, stereotactic biopsy is usually performed to confirm the diagnosis. Moreover, the subsequent chemotherapy standards vary for these two kinds of tumors.

In such a difficult situation, a powerful molecular imaging tool called ^18^F-FDG PET, which is expected to characterize the lesion on a metabolic and molecular level, is attractive. It is well known that FDG-uptake on diffuse large B-cell lymphoma is higher than that on other types of lymphoma, and this characteristic feature could be helpful in differentiating between B-cell lymphoma and other histological tumors [26]. Similarly, PET can identify degenerative diseases, multiple sclerosis, infectious diseases and cerebral infarction [27]. In recent years, the use of ^18^F-FDG PET and PET/CT has increased in routine practice at the time of diagnosis. Taking several studies together, this technique has a diagnostic sensitivity in PCNSL of 76%–100% [4, 28]. Maximum standardized uptake value (SUVmax) is higher in PCNSL than in gliomas [29, 30].

The first results of ^18^F-FDG PET in PCNSL were revealed by Rosenfeld et al. They found FDG-uptake in PCNSL lesions that were similar to lesions of anaplastic gliomas [31]. A few years afterwards, Palmedo et al. revealed that PCNSL lesions usually showed high FDG-uptake, which could be detected by ^18^F-FDG PET, with high sensitivity in immunocompetent patients [17]. Similarly, Kosaka et al. suggested that ^18^F-FDG PET had a reliable ability to differentiate between PCNSL and GBM when the cutoff value of SUVmax was 15 [16]. In agreement with the results of Kosaka et al., Makino et al. demonstrated a cutoff point of 12 [18]. However, Kawai et al. found the obvious advantage of PET to discriminate PCNSL from other tumorous and non-tumorous diseases in lesions with atypical MRI findings [19]. Although the sensitivity in the research of Kawai et al. was lower than that of the others, they noted that pretreatment FDG values could predict treatment response and tumor progression in patients with newly diagnosed PCNSL [32]. Overall, those studies were short of conviction, since the low incidence of PCNSL led to small sample size of study. The value of ^18^F-FDG PET and PET/CT in the diagnosis of PCNSL is still uncertain.

In this review, four studies used PET alone and three studies used PET/CT alone. Uniquely, in the study of Kosaka et al., both techniques were performed. Indeed, it should be noted that there are differences between PET and PET/CT. PET is a functional diagnostic imaging technique using compounds labeled with positron-emitting radioisotopes to measure cell metabolism [33]. In combination with CT, termed PET/CT, this method could detect lesion location more conspicuously. However, in a meta-analysis on the detection rate of ^18^F-FDG PET in marginal zone lymphoma of the mucosa-associated lymphoid tissue (MALT), there was no significant difference between the detection rate of PET/CT and PET alone [14]. Furthermore, we examined the heterogeneity between the eight studies that we included and no heterogeneity was found in the sensitivity and specificity of ^18^F-FDG PET and PET/CT in the patient-based data. Therefore, we put together two techniques in our study aiming to reveal the ability of ^18^F-FDG PET and PET/CT in finding disease, rather than finding the location of lesions.

In patients with PCNSL, compared with the immunocompetent population, the immunocompromised population often showed significant differences in clinical presentation, imaging feature, treatment and prognosis evaluation. Of note, in immunocompetent patients, necrosis and the resulting ring-enhancing lesions are rare. Conversely, imaging features are more variable in immunocompromised patients [34]. Additionally, in account of the better prognosis of the immunocompetent patients, our study mainly focused on assessing the value of ^18^F-FDG PET and PET/CT in the diagnosis of immunocompetent patients with PCNSL.

To the best of our knowledge, this is the first systematic review and meta-analysis aimed at revealing the diagnostic value of ^18^F-FDG PET and PET/CT in immunocompetent PCNSL patients. Overall, eight studies with 129 immunocompetent patients of PCNSL were included in the meta-analysis. In general, the results of our meta-analysis showed that ^18^F-FDG PET and PET/CT have a high diagnostic accuracy in patients with PCNSL. In regard to the patient-based analysis, the pooled sensitivity was 0.88 and the pooled specificity was 0.86. Moreover, the pooled PLR and NLR were 3.99 and 0.11, respectively. AUC presents the area under SROC, ranging from 0.5 to 1, which is used to evaluate the overall diagnosis effect. The larger the area is, the more powerful the detection ability of PET and PET/CT. In our meta-analysis, the AUC value was 0.9192. Q index is the point on the SROC curve at which the sensitivity and specificity are equal. Q index could assess the comprehensive diagnostic accuracy. In our meta-analysis, the Q index was 0.8525. Concerning these two parameters, our study indicated that ^18^F-FDG PET and PET/CT were of great value in the detecting of PCNSL in the immunocompetent population.

Indeed, it should be underlined that we included in this review the studies with patients who were suspected as PCNSL. ^18^F-FDG PET or PET/CT was applied to these patients as a radiological diagnostic tool. Based on the results of histopathology or clinical and radiologic follow-up, the TP, TN, FN and FP could be acquired or calculated. Unlike the ideal situation, ^18^F-FDG PET or PET/CT was used for PCNSL screening in the entire population. It could be attributed to that all of the included studies had adopt this criterion. In fact, the expense of ^18^F-FDG PET and PET/CT is great, as they are not suitable for cancer screening in the asymptomatic population. In this condition, although the specificity for diagnosing of PCNSL may be underestimated, the sensitivity would not be influenced. Still, we should have a clear understanding that PET cannot replace a surgical biopsy, which is essential for definitive diagnosis. Stereotactic biopsy is usually performed in a clinic to confirm the PCNSL diagnosis, which could compensate the lack of specificity in the initial diagnosis of ^18^F-FDG PET and PET/CT.

It is worth mentioning that, in regard to the pooled analysis, we only calculated the pooled sensitivity, specificity, PLR, NLR and DOR of the patient-based data (instead of the lesion-based data). This is mainly because most of the researchers of the included studies have adopted this method for data collection. Only in three studies would it have been possible to acquire the lesion-based data. The number of the lesions were 9, 16 and 34 for those three studies. The sample size is too small to conduct a pooled analysis. However, we should not ignore the potential diagnostic capability of ^18^F-FDG PET and PET/CT from the perspective of lesion-based analysis. It is hoped that future studies will focus on this issue.

Nevertheless, this systematic review and meta-analysis had some limitations. First, it is limited by the current existing literature. Although the diagnostic capability of ^18^F-FDG PET and PET/CT was well discussed in this review, it was not possible to assess the value of other aspects of disease management, such as staging, follow-up, and prognosis. Therefore, the clinical application value of ^18^F-FDG PET and PET/CT on PCNSL was not fully examined. Second, all of the studies were retrospective and the sample size was relatively small, which would weaken the confidence in making statistical conclusions. Further investigations were required to remedy this defect. In addition, since there were differences in observers’ experiences and the performance of instruments among the studies, potential measurement biases could exist.

Overall, based on current investigations, the findings of our meta-analysis demonstrate that ^18^F-FDG PET and PET/CT are valuable radiological diagnostic tools in immunocompetent PCNSL patients. They are conductive to narrowing the differential diagnosis for patients who were suspected as having PCNSL. Furthermore, they may provide useful information in addition to that obtained by MRI. We recommend ^18^F-FDG PET and PET/CT as appropriate choices for the routine diagnostic imaging method in PCNSL.

## MATERIALS AND METHODS

### Search strategy

Embase, PubMed/MEDLINE and Cochrane Library databases were searched based on the following strategy: (‘‘PET’’ OR ‘‘positron emission tomography’’) AND (‘‘primary central nervous system lymphoma’’ OR ‘‘primary CNS lymphoma’’ OR ‘‘PCNSL’’). The search was last updated on July 16^th^, 2016. There was no beginning date limit used. Reference lists of the included studies were also manually screened in before-mentioned databases in order to find the relevant additional study.

### Study selection

The inclusion criteria were: (1) Original articles revealing the performance of ^18^F-FDG PET or PET/CT in the initial diagnosis of PCNSL. (2) Studies in which the patients were diagnosed as having PCNSL, which was then confirmed by histopathology or clinical and radiologic follow-up. (3) Studies in which PET or PET/CT was used as the single reference standard for PCNSL diagnosis. (4) The most recent article or article with the most comprehensive information was included if the data from same patient were used in more than one article. (5) Articles with sufficient data to acquire or calculate the TP, FP, TN and FN. (6) Articles in which the full-text version could be acquired and articles that were published in the English language.

The exclusion criteria were: (1) Articles that cannot reveal the performance of ^18^F-FDG PET and PET/CT in the initial diagnosis of PCNSL. (2) Publications without primary data, such as comments, letters, case reports, conference proceedings, guidelines, and reviews. (3) *In vitro* studies and animal experiments. (4) Articles with patients who were in a situation of immunodeficiency or immunosuppression. (5) Articles with patients with diabetes mellitus. (6) Articles with less than five PCNSL patients enrolled. (7) Articles with patients who have been treated before (including steroid, surgical resection, chemotherapy, or radiotherapy). (8) Articles in which a full-text version could not be acquired or articles published in a non-English language.

### Data extraction and quality assessment

Independently, two investigators (Y.Z and J.T) reviewed the titles, abstracts and full-text articles, and extracted the data from the eligible studies. For each included study, basic information was collected concerning the study (name of the first authors, country of origin, year of publication, and study design), population characteristics of participants (number of subjects, sex and age distributions, immune states) and technical aspects of the study (imaging method used, SUVmax). In addition, the numbers of TP, FP, TN and FN findings for PET or PET/CT were recorded, as well as the method of PCNSL determination. Any disagreements would be resolved by discussion and a consensus.

The QUADAS-2 was used to estimate the quality of the included studies. This tool consists of four domains: patient selection, index test, reference standard and flow, and timing. Each domain involves the assessment of risk of bias (“low,” “high,” or “unclear”) and the applicability of diagnostic accuracy studies [35].

### Statistical analysis

Based on patient or lesion, the data of SUVmax, TP, FP, TN and FN were acquired or calculated from the primary data of each included study. For patient-based data, pool estimates of sensitivity, specificity, PLR, NLR and DOR were analyzed. Owing to no threshold effect existing, the Mantel–Haenszel method was performed to estimate the pooled data, which were presented as 95% confidence intervals. The SROC analysis was performed and plotted. The related AUC value and Q index were also estimated. Statistical analyses were executed using Stata software (version 13.0, StataCorp, College Station, Texas, USA). Meta-Disc software (version 1.4, Clinical Biostatistics Ramón y Cajal Hospital, Madrid, Spain) was adopted for drawing the plots and curve. The results of the statistical analysis were considered significant when the *p* value was < 0.05.
